# Interstitial lung disease incidence and mortality in the UK and the European Union: an observational study, 2001–2017

**DOI:** 10.1183/23120541.00058-2022

**Published:** 2022-07-11

**Authors:** Justin D. Salciccioli, Dominic C. Marshall, Richard Goodall, Conor Crowley, Joseph Shalhoub, Preya Patel, Philip L. Molyneaux

**Affiliations:** 1 Pulmonary and Critical Care Medicine, Brigham and Women's Hospital, Harvard Medical School, Boston, MA, USA; 2 Dept of Surgery and Cancer, Imperial College London, London, UK; 3 Medical Data Research Collaborative, London, UK; 4 Guy's and St Thomas’ NHS Foundation Trust, London, UK; 5 Oxford University Hospitals NHS Foundation Trust, Oxford, UK; 6 Dept of Critical Care, Lahey Hospital and Medical Center, Burlington, MA, USA; 7 Imperial College Healthcare NHS Trust and Imperial College London, London, UK; 8 Dept of Internal Medicine, The Wright Center, Scranton, PA, USA; 9 Royal Brompton and Harefield Hospitals, Guy's and St Thomas’ NHS Foundation Trust London, London, UK

## Abstract

**Objective:**

To compare the trends in age-standardised incidence and mortality from interstitial lung diseases (ILD) in the UK and the European Union (EU).

**Methods:**

This was an observational study using data obtained from the Global Burden of Disease Study on residents of the UK and of the 27 EU countries. The main outcome measures were ILD age-standardised incidence rates per 100 000 (ASIR), age-standardised death rates per 100 000 (ASDR) and mortality-to-incidence ratios (MIRs), which are presented for men and women separately for each country for the years 2001–2017. Trends were analysed using joinpoint regression analysis.

**Results:**

In 2017, the median incidence of ILD was 7.22 (IQR 5.57–8.96) per 100 000 population for men and 4.34 (IQR 3.36–6.29) per 100 000 population for women. In 2017, the median ASDR attributed to ILD was 2.04 (IQR 1.13–2.71) per 100 000 population for men and 1.02 (0.68–1.37) per 100 000 population for women. There was an overall increase in ASDR during the observation period, with a median increase of +20.42% (IQR 5.44–31.40) for men and +15.44% (IQR −1.01–31.52) for women. Despite increases in mortality over the entire observation period, there were decreasing mortality trends in the majority of countries at the end of the observation period (75% for men and 86% for women).

**Conclusion:**

Over the past two decades, there have been increases in the incidence and mortality of ILD in Europe. The most recent trends, however, demonstrate decreases in mortality from ILD in the majority of European countries for both men and women. These data support the ongoing improvements in the diagnosis and management of ILD.

## Abstract 2

**Recent improvements in the diagnosis and management of interstitial lung disease, as well as the introduction of therapeutic agents, have resulted in significant decreases in mortality in the majority of European countries** https://bit.ly/3JHkEyO

## Introduction

Interstitial lung disease (ILD) is a group of heterogeneous diseases with a common feature of damage to lung parenchyma and alveoli. It is characterised by inflammation and fibrosis [1]. ILD is associated with systemic inflammatory diseases such as rheumatoid arthritis and sarcoidosis, and may also be associated with environmental or occupational exposures. In the majority of ILD cases, however, a specific aetiological factor is never identified. The last two decades have seen changes in therapies for ILD, particularly for idiopathic pulmonary fibrosis (IPF), in which some therapies demonstrated harm [2], and two novel anti-fibrotic agents have been licensed [3–5].

Estimates of incidence and mortality attributed to ILD have increased in recent years but remain limited, in part due to the multiple aetiological factors and difficulty in diagnosis, as well as geographical differences in diagnostic criteria and thresholds [6]. Previous reports on ILD incidence in Europe include registries and questionnaires [7–9]. One recent review assessed the incidence of fibrosing-type in IPF and ILD globally and found that there is a paucity of data on the incidence of this heterogeneous group of diseases, with substantial variation between health systems [10]. We have previously analysed mortality from IPF across the European Union (EU) using the World Health Organization (WHO) mortality database, which includes mandatory reporting of cause of death, and found substantial variation in mortality between countries [11]. However, there was no similar widespread registry data for disease incidence and, as such, an up-to-date analysis of ILD incidence and mortality across the UK and EU is warranted.

The objective of this study was to describe current incidence and mortality rates as well as overall trends in ILD in the UK and EU using the Global Burden of Disease (GBD) study results. These data are collated by the GBD Collaborators and made available publicly for analysis. Given the increasing recognition of ILD as an important cause of morbidity and mortality, and the increasing case identification, our hypothesis was that there would be increasing trends in both incidence and mortality from ILD across the UK and EU. We analysed trends in ILD incidence and mortality between 2001 and 2017 using joinpoint regression analysis.

## Methods

### Data source

The data for this observational analysis of ILD were obtained from the GBD database, which collates mortality and disability data (deaths, death rates, years of life lost due to premature mortality, prevalence and incidence) for a collection of global health concerns. The exact GBD methodology has been published previously [12] and we have used the GBD source previously in reports relating to abdominal aortic aneurysm [13] and peripheral arterial disease [14]. Briefly, the GBD uses systematic reviews, survey data, disease registries, hospital administrative data, claims, inpatient and outpatient data, and case notifications as data sources to estimate disease incidence. Disease classifications are based on the International Classification of Disease (ICD) coding system (9th and 10th revisions). The data are collated by the GBD Collaborators and made available publicly for analysis.

Cases were defined as a combined output of ILD including pulmonary sarcoidosis using the ICD-10 codes J84 and D86, respectively. Previous reports on ILD have included sarcoidosis in this definition and have been reported by the GBD investigators [15, 16]. Incidence data were sourced from literature reviews, claims data (for the United States of America, not required in our study) and hospital inpatient records. Using these data as input, incidence estimates were computed using a standard strategy with parameters described previously [17].

Mortality data are collected primarily from seven sources (vital registration, verbal autopsy, cancer registry, police records, sibling history, surveillance and survey/census). The produced information is available to the public and can be extracted *via* the GBD Results Tool (http://ghdx.healthdata.org/gbd-results-tool). We used this tool to extract age-standardised incidence and mortality rates for ILD for the UK and EU countries between 2001 and 2017. Mortality was also reported as a combined value for ILD and pulmonary sarcoidosis. Owing to the manner in which data are stored in the GBD study, we were unable to separate sarcoidosis from other aetiologies of ILD for the purposes of this analysis. Input for estimates was sourced from vital registration and surveillance data from the cause of death database. These data were filtered using the following exclusion criteria: values implausibly high or low, significant conflict with established age or temporal patterns, and significant conflict with other sources of data for the same or similar regions. Input data were used to compute mortality estimates using the standard GBD modelling described in detail by the GBD Collaborators [17].

### Handling of the GBD data

Age-standardised incidence rates (ASIRs) and age-standardised death rates (ASDRs) for ILD stratified by sex and age-standardised per 100 000 population were extracted from the GBD Results Tool for each of the years between 2001 and 2017, inclusive, for the UK and EU countries. Extracting age-standardised rates improves inter-country comparability, because it accounts for differences in the age structure of different populations. For all age-standardised rates, the GBD study computes a standard population using a non-weighted average across a percentage of the population of all countries in each 5-year age bracket (years 2010–2035) from the United Nations Population Division's World Population Prospects (2012 revision [18]).

Absolute and relative changes in ASIRs and ASDRs over the observation period (*i.e.* differences between the rates in 2001 and 2017) were calculated for each sex in each country. ASDRs were quantified as a proportion of ASIRs by dividing ASDR by the ASIR to calculate a mortality-to-incidence ratio (MIR) for each year (2001–2017, inclusive) for each sex in each country. MIRs have previously been shown to correlate well with cancer management outcomes, and their use can help us to understand how the impact and management of ILD has varied temporally with sex and location. The MIR represents the case-fatality rate, and is calculated by dividing the mortality count by incidence per annum for a specific population. This allows for comparisons between different geographical locations by standardising to local incidence rates and is useful for understanding survival and burden of disease because it provides an estimate of regional case-fatality. A low MIR means there is lower mortality of the condition in relation to incidence and a high MIR equates to higher mortality in relation to the incidence in that population. The MIR has been used in multiple previous investigations and we have previously used this metric to assess survival in other populations [13, 14].

The GBD quantifies the availability and completeness of the mortality data by each location-year to indicate the reliability of cause of death data. Each country is graded on a 5-star scale. For the countries analysed in the present analysis, with the exceptions of Cyprus and Slovakia (2 stars and 3 stars, respectively), 15 EU countries scored 4 stars (Belgium, Bulgaria, Croatia, Czech Republic, Denmark, France, Germany, Greece, Luxembourg, Netherlands, Poland, Portugal, Romania, Slovenia and Spain), representing >65% completeness of mortality data. The UK and the 10 remaining EU countries (Austria, Estonia, Finland, Hungary, Ireland, Italy, Latvia, Lithuania, Malta and Sweden) have 5-star data, demonstrating >85% completeness of the data. In addition to the above analysis for the UK and 27 EU member states, we performed a secondary *post hoc* analysis of the 5-star countries (supplementary figures S1–S4).

### Statistical analysis

Joinpoint regression analysis was used to assess trends in the disease burden of ILD. The joinpoint software (Joinpoint Command Line Version 4.5.0.1) was provided by the United States National Cancer Institute Surveillance Research Program [19]. This software tracks trends in data over time (for the present analysis, ASIRs and ASDRs), then fits the simplest model possible to the data by connecting several different line segments on a logarithmic scale. These segments are known as joinpoints, with the simplest model (*i.e.* 0 joinpoints) being a straight line. As more joinpoints are added, each is tested for significance using a Monte Carlo permutation method. The software also gives estimated annual per cent changes (EAPCs) for each line segment (with corresponding 95% confidence intervals). Each EAPC is tested to establish if a difference from the null hypothesis of no change exists. Consequently, the final model consists of multiple joinpoints, each representing a statistically significant (p<0.05) change in trend (increase or decrease), with each trend described by the EAPC and the associated confidence intervals. The EAPC allows trend changes to be assessed at a constant per cent per year.

## Results

A total of 28 countries were included in this investigation, including the UK as well as the 27 EU member states of Austria, Belgium, Bulgaria, Croatia, Cyprus, the Czech Republic, Denmark, Estonia, Finland, France, Germany, Greece, Hungary, Ireland, Italy, Latvia, Lithuania, Luxembourg, Malta, Netherlands, Poland, Portugal, Romania, Slovakia, Slovenia, Spain and Sweden.

### Current estimates of ILD incidence

**FIGURE 1:**
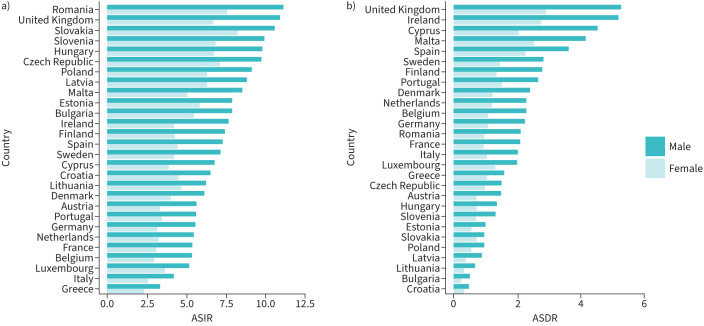
a) Age-standardised incidence rates (ASIRs) and b) age-standardised death rates (ASDRs) per 100 000 population for interstitial lung diseases for men and women in the UK and Europe in 2017.

A summary of current estimates of ILD incidence is shown in figure 1a. For men in 2017, the median incidence of ILD was 7.22 (interquartile range (IQR) 5.57–8.96) per 100 000 population. The countries with the highest ASIRs of ILD for men in 2017 included Romania (11.14), the UK (10.92), Slovakia (10.58) and Slovenia (9.93). The countries with the lowest ASIRs for men in 2017 included Greece (3.30), Italy (4.18), Luxembourg (5.15) and Belgium (5.33). For women in 2017, the median incidence of ILD was 4.34 (IQR 3.36–6.29) per 100 000 population. The countries with the highest ASIRs in 2017 for women included Slovakia (8.25), Romania (7.52), the Czech Republic (7.10) and Slovenia (6.83). The countries with the lowest ASIRs for women included Greece (2.26), Italy (2.53), Belgium (2.96) and France (3.08).

### Current estimates of ILD mortality

A summary of the current estimates of ILD mortality is shown in figure 1b. Overall, the median ASDRs attributed to ILD in 2017 were 2.04 (IQR 1.13–2.71) and 1.02 (0.68–1.37) per 100 000 population, for men and women respectively. The countries with the highest ASDRs for men in 2017 included the UK (5.27), Ireland (5.18), Cyprus (4.52) and Malta (4.14). The countries with the lowest ASDRs for men in 2017 included Croatia (0.44), Bulgaria (0.47), Lithuania (0.64) and Latvia (0.86). The countries with the highest ASDRs for women in 2017 included the UK (2.89), Ireland (2.73), Malta (2.51) and Spain (2.25). The countries with the lowest ASDRs for women in 2017 included Bulgaria (0.18), Croatia (0.26), Lithuania (0.26) and Latvia (0.33).

### Current estimates of ILD MIRs

The median MIRs were 0.38 (IQR 0.13–0.47) and 0.31 (IQR 0.10–0.42) for men and women, respectively. The countries with the highest MIRs for men in 2017 were Ireland (0.68), Cyprus (0.67), Spain (0.50) and Malta (0.49). The countries with the lowest MIRs for men in 2017 were Bulgaria (0.06), Croatia (0.07), Slovakia (0.09) and Latvia (0.10). The countries with the highest MIRs for women in 2017 were Ireland (0.65), Spain (0.51), Cyprus (0.50) and Malta (0.50). The countries with the lowest MIRs for women in 2017 included Bulgaria (0.03), Latvia (0.05), Lithuania (0.06) and Croatia (0.06).

### Changes in ILD incidence between 2001 and 2017

**FIGURE 2:**
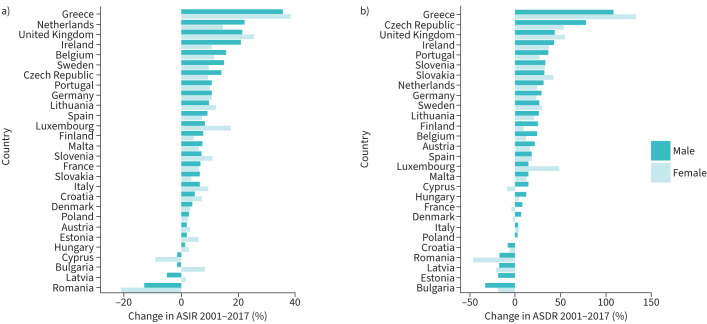
Changes in a) age-standardised incidence rates (ASIRs) and b) age-standardised death rates (ASDRs) for interstitial lung diseases for men and women in the UK and Europe.

For men, there was an overall increase in the incidence of ILD with a median change of +7.14% (IQR 2.23–12.29%) (figure 2a). The incidence of ILD increased across all countries except for Romania (−12.95%), Latvia (−4.94%), Bulgaria (−1.48%) and Cyprus (−1.30%), which all had overall decreases in the incidence of ILD for men. The greatest increases were in Greece (+35.51%), the Netherlands (+22.03%), the UK (+21.27%) and Ireland (+20.79%). A summary of the changes in incidence rates for men is shown in table 1. For women, there was an overall increase in the incidence of ILD with a median change of +7.66% (IQR 3.29–10.70%). There was an increase in ILD incidence for women in all countries except for Romania (−20.76%) and Cyprus (−9.11%). The greatest increases were in Greece (+38.36%), the UK (+25.39%), Luxembourg (+17.28%) and the Netherlands (+14.64%). A summary of the changes in incidence rates for women is shown in table 2.

**TABLE 1:** Male age-standardised incidence rates (ASIRs) and age-standardised death rates (ASDRs) per 100 000 population between 2001 and 2017, and percentage change over the observation period

	**Incidence (ASIR per 100 000)**			**Mortality (ASDR per 100 000)**		
**Country**	**Start**	**End**	**Change (%)**	**Start**	**End**	**Change (%)**
**Austria**	5.51	5.61	0.1 (1.88)	1.19	1.46	0.27 (22.27)
**Belgium**	4.61	5.33	0.72 (15.56)	1.81	2.26	0.45 (25.02)
**Bulgaria**	7.97	7.85	−0.12 (−1.48)	0.7	0.47	−0.23 (−32.38)
**Croatia**	6.22	6.51	0.28 (4.54)	0.48	0.44	−0.03 (−7.08)
**Cyprus**	6.86	6.77	−0.09 (−1.3)	3.94	4.52	0.58 (14.64)
**Czech Republic**	8.55	9.74	1.19 (13.89)	0.83	1.47	0.64 (78)
**Denmark**	5.93	6.15	0.22 (3.72)	2.24	2.4	0.16 (7.09)
**Estonia**	7.74	7.88	0.14 (1.81)	1.18	0.96	−0.22 (−18.63)
**Finland**	6.88	7.4	0.52 (7.53)	2.2	2.76	0.56 (25.52)
**France**	5.04	5.37	0.33 (6.59)	1.91	2.07	0.16 (8.35)
**Germany**	5.03	5.57	0.53 (10.62)	1.72	2.22	0.5 (28.74)
**Greece**	2.43	3.3	0.86 (35.51)	0.75	1.55	0.81 (108.1)
**Hungary**	9.7	9.82	0.12 (1.22)	1.19	1.34	0.15 (12.69)
**Ireland**	6.35	7.66	1.32 (20.79)	3.62	5.18	1.56 (43.19)
**Italy**	3.93	4.18	0.25 (6.26)	1.94	2.01	0.07 (3.79)
**Latvia**	9.27	8.81	−0.46 (−4.94)	1.04	0.86	−0.17 (−16.72)
**Lithuania**	5.67	6.21	0.54 (9.44)	0.51	0.64	0.13 (26.01)
**Luxembourg**	4.76	5.15	0.38 (8)	1.71	1.97	0.26 (15.17)
**Malta**	7.93	8.51	0.58 (7.3)	3.61	4.14	0.53 (14.81)
**Netherlands**	4.46	5.44	0.98 (22.03)	1.73	2.26	0.53 (30.9)
**Poland**	8.88	9.11	0.23 (2.58)	0.88	0.91	0.03 (3.15)
**Portugal**	5.03	5.57	0.54 (10.69)	1.95	2.65	0.71 (36.28)
**Romania**	12.8	11.14	−1.66 (−12.95)	2.5	2.08	−0.41 (−16.56)
**Slovakia**	9.95	10.58	0.63 (6.34)	0.7	0.93	0.22 (31.9)
**Slovenia**	9.28	9.93	0.65 (6.98)	0.97	1.29	0.32 (33.42)
**Spain**	6.69	7.29	0.6 (9.01)	3.05	3.61	0.57 (18.56)
**Sweden**	6.22	7.14	0.92 (14.84)	2.21	2.81	0.6 (27.2)
**UK**	9	10.91	1.91 (21.27)	3.67	5.27	1.6 (43.49)

**TABLE 2:** Female age-standardised incidence rates (ASIRs) and age-standardised death rates (ASDRs) per 100 000 population between 2001 and 2017, and percentage change over the observation period

	**Incidence (ASIR per 100 000)**			**Mortality (ASDR per 100 000)**		
**Country**	**Start**	**End**	**Change (%)**	**Start**	**End**	**Change (%)**
**Austria**	3.24	3.33	0.09 (2.91)	0.58	0.68	0.1 (17.49)
**Belgium**	2.65	2.96	0.3 (11.47)	0.93	1.05	0.12 (12.32)
**Bulgaria**	5.04	5.45	0.41 (8.2)	0.22	0.18	−0.04 (−17.57)
**Croatia**	4.16	4.45	0.29 (7.04)	0.28	0.26	−0.01 (−5.4)
**Cyprus**	4.28	3.89	−0.39 (−9.11)	2.13	1.96	−0.18 (−8.21)
**Czech Republic**	6.51	7.1	0.6 (9.14)	0.63	0.96	0.33 (53.4)
**Denmark**	3.84	3.96	0.12 (3.13)	1.21	1.19	−0.01 (−1.17)
**Estonia**	5.51	5.82	0.31 (5.65)	0.53	0.53	0 (0.41)
**Finland**	4.07	4.24	0.17 (4.24)	1.21	1.32	0.11 (9.37)
**France**	2.91	3.08	0.18 (6.04)	0.93	0.91	−0.02 (−2.48)
**Germany**	2.84	3.14	0.3 (10.44)	0.86	1.06	0.2 (23.44)
**Greece**	1.63	2.26	0.63 (38.36)	0.44	1.02	0.58 (132.61)
**Hungary**	6.56	6.73	0.17 (2.56)	0.64	0.68	0.03 (4.79)
**Ireland**	3.81	4.2	0.4 (10.49)	1.99	2.73	0.74 (37.38)
**Italy**	2.32	2.53	0.22 (9.28)	0.98	1.03	0.05 (4.63)
**Latvia**	6.22	6.31	0.09 (1.47)	0.41	0.33	−0.08 (−20.48)
**Lithuania**	4.19	4.69	0.5 (11.83)	0.22	0.26	0.05 (20.83)
**Luxembourg**	3.08	3.61	0.53 (17.28)	0.87	1.29	0.43 (49.43)
**Malta**	4.76	5.05	0.29 (6.1)	2.21	2.51	0.3 (13.39)
**Netherlands**	2.8	3.21	0.41 (14.64)	0.95	1.18	0.23 (24.63)
**Poland**	6.12	6.26	0.14 (2.26)	0.52	0.52	0 (−0.84)
**Portugal**	3.08	3.39	0.31 (10.23)	1.18	1.5	0.32 (26.84)
**Romania**	9.49	7.52	−1.97 (−20.76)	1.7	0.92	−0.77 (−45.66)
**Slovakia**	7.98	8.25	0.28 (3.46)	0.49	0.7	0.21 (42.03)
**Slovenia**	6.17	6.84	0.67 (10.92)	0.51	0.68	0.17 (32.87)
**Spain**	4.15	4.44	0.3 (7.13)	1.89	2.25	0.36 (19.32)
**Sweden**	3.85	4.22	0.37 (9.51)	1.09	1.41	0.33 (30.17)
**UK**	5.35	6.7	1.36 (25.39)	1.87	2.89	1.02 (54.56)

### Changes in ILD mortality between 2001 and 2017

**FIGURE 3:**
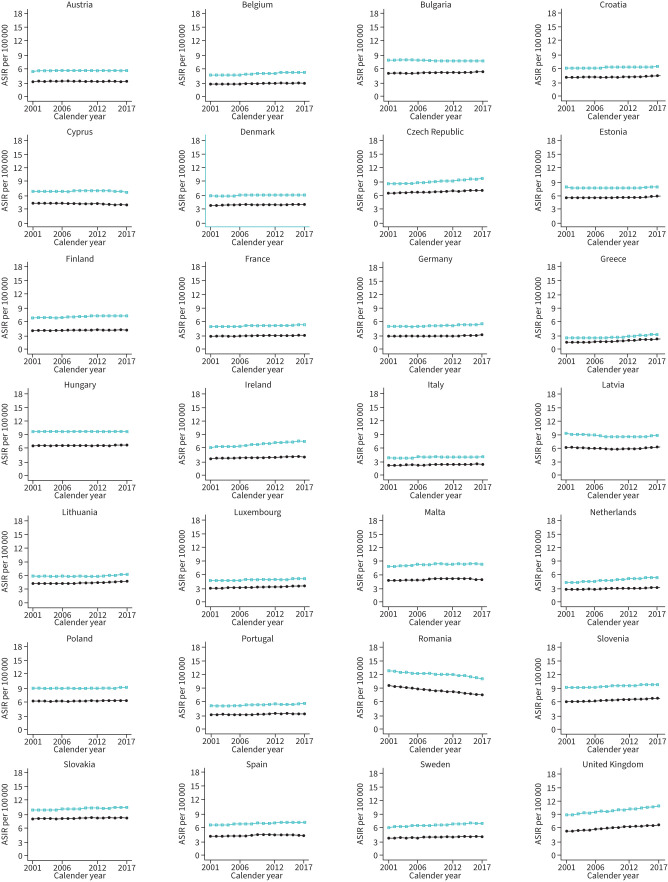
Trends in age-standardised incidence rates (ASIRs) per 100 000 for interstitial lung diseases across the UK and Europe. Green squares represent men and solid circles represent women.

For men, there was an overall increase in ASDR during the observation period with a median change of +20.42% (IQR 5.44–31.40%) (figure 3b). The ASDRs for ILD increased in all countries except Bulgaria (−32.38%), Estonia (−18.63%), Latvia (−16.72%), Romania (−16.57%) and Croatia (−7.09%). The greatest increases were in Greece (+108.1%), the Czech Republic (+78.02%), the UK (+43.49%) and Ireland (+43.19%). A summary of the changes in male mortality from ILD is shown in table 1. For women, there was an overall increase in ASDR during the observation period with a median increase of +15.44% (IQR −1.01–31.52%). There were overall decreases in ILD mortality in women in Romania (−45.66%), Latvia (−20.48%), Bulgaria (−17.57%), Cyprus (−8.21%), Croatia (−5.40%), France (−2.47%), Denmark (−1.17%) and Poland (−0.84%). All other countries had increasing mortality in women, with the greatest increases observed in Greece (+132.61%), the UK (+54.56%), the Czech Republic (+53.40%) and Luxembourg (+49.43%). A summary of the changes in female mortality from ILD is shown in table 2.

### Changes in ILD MIRs between 2001 and 2017

The MIR for men increased over the observation period with a median change of +9.76% (IQR 1.10–18.43%). There were decreasing MIRs in Bulgaria (−31.37%), Estonia (−20.08%), Latvia (−12.39%), Croatia (−11.12%), Romania (−4.15%) and Italy (−2.32%). The countries with the greatest percentage increase in MIRs were the Czech Republic (+56.29%), Greece (+53.56%), Slovenia (+24.72%) and Slovakia (+24.04%). For women, there was an overall MIR increase of +7.46% (IQR −4.22–19.33%). The countries with the greatest decreases in MIR for women were Romania (−31.42%), Bulgaria (−23.82%), Latvia (−21.63%) and Croatia (−11.62%). The countries with the greatest increases in MIR for women were Greece (+68.12%), the Czech Republic (+40.55%), Slovakia (+37.28%) and Luxembourg (+27.42%).

### Joinpoint trends for ILD incidence

The results of joinpoint regression analysis for incidence are shown in figure 3 (and supplementary tables S1 and S2). In men, the greatest decreases in EAPCs were observed in Latvia between 2005 and 2009 (EAPC −1.5, 95% CI −1.7– −1.3) and in Romania between 2014 and 2017 (EAPC −1.3, 95% CI −1.4– −1.3). The greatest increases in EAPCs for men were observed in Greece between 2006 and 2010 (EAPC +2.0, 95% CI 1.9–2.1), between 2010 and 2013 (EAPC +2.4, 95% CI 2.2–2.6) and between 2013 and 2017 (EAPC +3.0, 95% CI 3.0–3.1). For women, decreasing EAPCs were observed in Romania between 2001 and 2003 (EAPC −1.6, 95% CI −1.6– −1.6) and in Cyprus between 2014 and 2017 (EAPC −1.6, 95% CI −1.8– −1.4). The greatest increases in EAPCs for women were in Greece between 2006 and 2014 (EAPC +2.6, 95% CI 2.5–2.6) and between 2014 and 2017 (EAPC +2.8, 95% CI 2.6–3.0).

### Joinpoint trends for ILD mortality

**FIGURE 4:**
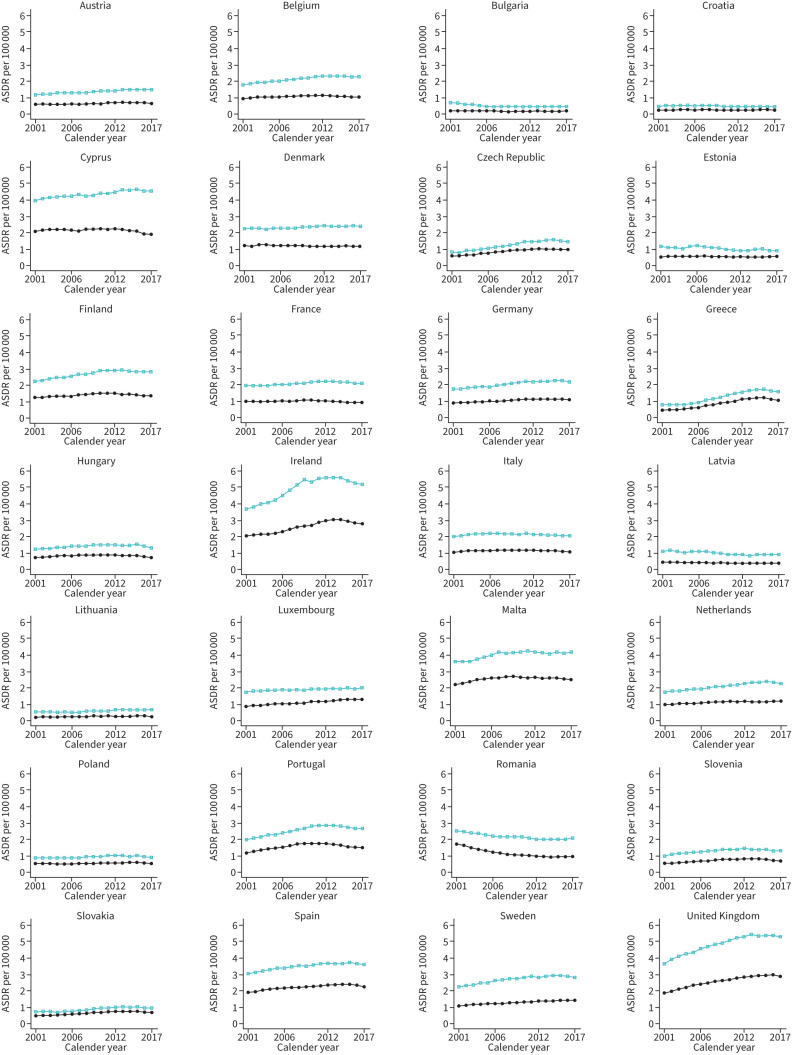
Trends in age-standardised death rates (ASDRs) per 100 000 for interstitial lung diseases across the UK and Europe. Green squares represent men and solid circles represent women.

The results of joinpoint regression analysis for mortality are shown in figure 4 (and supplementary tables S3 and S4). For men, trends in ASIRs varied between countries and the greatest negative EAPCs were observed in Bulgaria between 2001 and 2006 (EAPC −6.7, 95% CI −7.3– −6.2) and in Slovakia and Hungary between 2015 and 2017 (EAPC −5.1, 95% CI −8.1– −2.1 and EAPC −4.5, 95% CI −6.6– −2.4, respectively). For women, the greatest decreasing EAPCs were in Greece between 2015 and 2017 (EAPC −7.8, 95% CI −13.8– −1.3) and in Hungary between 2015 and 2017 (EAPC −7.3, 95% CI −9.5– −5.1). For men, the greatest increases in EAPC were in Greece between 2005 and 2011 (EAPC +10.4, 95% CI 8.9–12.0) and in Czech Republic between 2001 and 2012 (EAPC +5.7, 95% CI 5.4–6.0). For women, the greatest increases in EAPCs were in Greece between 2001 and 2004 (EAPC +6.4, 95% CI 2.9–10.0) and between 2004 and 2012 (EAPC +9.6, 95% CI 8.6–10.6). Despite overall increases in EAPC for mortality over the entire observation period, there were decreasing mortality trends in the majority of countries at the end of the observation period (21 of 28 (75%) for men and 24 of 28 (86%) for women; supplementary tables S3 and S4).

### Joinpoint trends for ILD MIRs

The results of joinpoint regression for MIRs for men and women are shown in supplementary figure S2 and summarised in supplementary tables S5 and S6. For men, the greatest decreases in EAPCs were in Greece between 2015 and 2017 (EAPC −8.7, 95% CI −14.2– −2.8) and Bulgaria between 2001 and 2006 (EAPC −6.7, 95% CI −7.2– −6.1). The greatest increases in MIR EAPCs for men were in Greece between 2005 and 2011 (EAPC +8.4, 95% CI 6.9–10.0) and in Slovenia between 2001 and 2003 (EAPC +7.2, 95% CI 3.1–11.4). For women, the greatest decreases in MIR were observed in Greece between 2015 and 2017 (EAPC −10.3, 95% CI −15.6– −4.6), in Croatia between 2015 and 2017 (EAPC −7.6, 95% CI −14.6– −0.1) and in Hungary between 2015 and 2017 (EAPC −7.4, 95% CI −9.7– −5.0). The greatest increases in EAPCs for women were observed in Greece between 2001 and 2012 (EAPC +7.0, 95% CI 6.6–7.4) and in Hungary between 2001 and 2004 (EAPC +6.2, 95% CI 4.9–7.6). Despite overall increasing MIR over the entire observation period, there were decreasing MIR trends in the majority of countries at the end of the observation period (23 of 28 (82%) for both men and women; supplementary tables S5 and S6).

## Discussion

### Principal findings

In this observational study of ILD across the EU and UK between 2001 and 2017, we have shown an overall increasing incidence of ILD across most countries for both men and women. In most countries, men have greater incidence and mortality compared to women, a difference which persists throughout the observation period. Further, despite overall increases in mortality between 2001 and 2017, there have been decreases in mortality for men and women in the majority of countries at the end of the observation period. The MIR of ILD is flattening or decreasing in most countries, with negative trends in MIR for men and women in the majority of countries in Europe.

The principal aim of this investigation was to obtain current estimates of the burden of ILD across Europe. Specifically, we sought to understand the changes in incidence and mortality over the past two decades because this time period has seen much development in the classification, diagnosis and management of various ILDs. Providing a comprehensive look at incidence and mortality from ILD across Europe, this report will serve as a useful benchmark for current burden of ILD and for monitoring future progress in the management of this heterogeneous group of chronic lung diseases.

### Comparison with prior studies

The majority of reports to date have attempted to provide estimates of incidence and mortality in ILDs utilising primary physician surveys, large health system databases or surveys of respiratory physicians. These previous reports have focused on single health systems and are unable to make strong comparisons in incidence and mortality between countries or health systems. For instance, Kornum *et al*. [20] performed an analysis of a national health database in Denmark and reported adjusted incidence rates of ILD of approximately 2.91 per 100 000 population while another study from the same country reported annual incidence of 4.1 per 100 000 population cohort [21]. These estimates are similar to the incidence estimated in other health systems including Greece, which had a reported incidence of 4.63 per 100 000 population [22], and significantly higher than the incidence reported recently in France at 1.94 per 100 000 population [23]. These data highlight significant differences in estimates of incidence of ILDs between health systems and no prior study has attempted to make direct comparisons between countries as we have done in the current report.

Previously, our research group assessed mortality from IPF across Europe [11]. We demonstrated that across most of the EU, the mortality from IPF was increasing between 2001 and 2014. This report was limited, however, in that we were unable to estimate incidence rates and it was not possible to demonstrate whether increases in mortality from IPF were related to increased awareness and identification of IPF, or whether mortality was increasing independent of incidence. One previous report has also shown increasing incidence and prevalence of IPF in the UK over a similar period [24]. As a result, we planned the current study to better estimate both incidence and mortality from ILD (inclusive of IPF) and to estimate the changes in mortality relative to changing incidence over a similar observation period. Here, our data suggest that over the past two decades, there have been marginal increases in the overall incidence of ILD and greater increases in mortality during the time period.

Despite this, we observed significant decreasing trends in ILD mortality and in MIRs in multiple countries during the most recent 5 years of observation. Specifically, we observed statistically significant negative trends in mortality for 21 countries (75%) for men and in 24 countries (86%) for women for trends ending in 2017. Further, using MIR to understand recent management of this chronic lung disease, we demonstrated that there were significant decreasing trends in MIR in 23 countries (82%) for men and in 23 countries (82%) for women. Although our study assessed ILD broadly, this finding is important because there have been advances in the treatment of IPF with the introduction of anti-fibrotic agents in the EU from as early as 2012 [3, 4]. The approval of anti-fibrotic agents was a significant event in the treatment of IPF because, for the first time, therapeutic agents were available to slow the progression of lung fibrosis and reduce mortality. Although our data are observational in nature and we are unable to make causal statements, IPF constitutes a significant portion of ILD diagnoses with estimates of nearly 20% of all ILDs related to IPF [2], and our study suggests decreases in mortality from ILD after the introduction of anti-fibrotic agents in multiple health systems. Future investigations should, therefore, aim to clarify whether the observed decreases in mortality from ILD are, in fact, driven by improved management of IPF during this period. Furthermore, while no one particular feature has been identified as causal in the development of ILD including sarcoidosis, there are multiple potential explanations for the between-country differences observed in our study. Previous reports of sarcoidosis, for example, have highlighted geographic differences related to sarcoidosis incidence which are potentially associated with geographic features including higher latitudes and sunlight exposure, exposure to coastal or rural areas, and agricultural employment or exposure to environmental antigens. Other ILDs may have other associated risk factors: *e.g.* hypersensitivity pneumonitis may have greater burden in areas with high agricultural employment, and IPF has been associated with greater exposure to environmental particulates (*e.g.* particles with a 50% cut-off aerodynamic diameter of 2.5 µm), as well as having strong associations with lifestyle factors such as tobacco smoke and with other comorbid conditions such as gastro-oesophageal reflux disease. Our study was not designed to assess each of these potential contributors and although it is unlikely that any one of these additional variables is the single explanatory factor for the observed differences, we have highlighted these additional potential variables as future work may help to elucidate the individual relationships with ILD morbidity and mortality across Europe.

### Strengths and weaknesses of the study

The major strengths of this report are the total number of countries observed and the total duration of the observation period for analysis. We used standardised estimates of incidence and mortality, which allows us to make comparisons between countries by removing the influence of country-specific demographics on these variables. Most previous reports have focused on the epidemiology of ILD within a single health system or with shorter observation periods. Furthermore, we have also used MIR as a marker of performance in management of ILD.

Despite these strengths, there are several limitations that must be considered when interpreting the results of this observational study. First, the data attained are applicable solely for the purpose of identification and comparison of ILD between the EU countries and the UK and, as such, causal relationships cannot be drawn. We acknowledge that confounding variables beyond the scope of discussion will have differential effects by country on the data presented from this observational study: using sex-specific, age-standardised mortality and incidence rates attempts to account for some confounding of demography. Next, our data represent the broad category of ILD, which comprises diseases with heterogeneous clinical features and diagnosis, with variations in management and prognosis. Individuals with sarcoidosis, for example, may carry the diagnosis through lymph node biopsy but lack pulmonary involvement until late stages (*i.e.* stage IV) of the disease. Owing to the manner in which GBD reports their data, we are unable to ascertain specific features of trends relating to specific causes of ILD such as IPF, and by including sarcoidosis we may potentially underestimate the true mortality burden from other ILDs. Future work should attempt to characterise trends in individual ILD aetiologies. As outlined above, there is variation in the reporting of data quality between European countries that may also influence the results of this study. We attempted to address this limitation by performing a sub-group analysis with data restricted to those countries with a 5-star rating in order to improve comparability (supplementary material).

### Conclusion

Over the past two decades, there have been increases in the incidence and mortality of ILD in Europe. Recent trends, however, demonstrate decreases in mortality from ILD in the majority of European countries for both men and women. These data support the ongoing improvements in the diagnosis and management of ILD.

## Supplementary material

10.1183/23120541.00058-2022.Supp1 **Please note:** supplementary material is not edited by the Editorial Office, and is uploaded as it has been supplied by the author.
Supplementary material 00058-2022.SUPPLEMENT
