# Supplementary material for: Interstitial lung disease incidence and mortality in the UK and the European Union: an observational study, 2001–2017
Source: ERJ Open Res. 2022 Jul 11;8(3):00058-2022. doi: 10.1183/23120541.00058-2022 (PMC9271755; doi:10.1183/23120541.00058-2022)
Supplement: Supplementary file 1 [file 00058-2022.SUPPLEMENT.pdf]

Supplementary materials

Supplementary figure legends

Supplementary figure S1: Trends in mortality-to-incidence ratios for interstitial lung disease across Europe. White circles represent male and solid squares represent female.

Supplementary figure S2: Trends in incidence for interstitial lung disease for European countries with high (5-star) data quality rating. White circles represent male and solid squares represent female.

Supplementary figure S3: Trends in mortality for interstitial lung disease for European countries with high (5-star) data quality rating. White circles represent male and solid squares represent female.

Supplementary figure S4: Trends in DALYs for interstitial lung disease for European countries with high (5-star) data quality rating. White circles represent male and solid squares represent female.

Supplementary figure S5: Trends in MIR for interstitial lung disease for European countries with high (5-star) data quality rating. White circles represent male and solid squares represent female.

Supplementary figure S1:

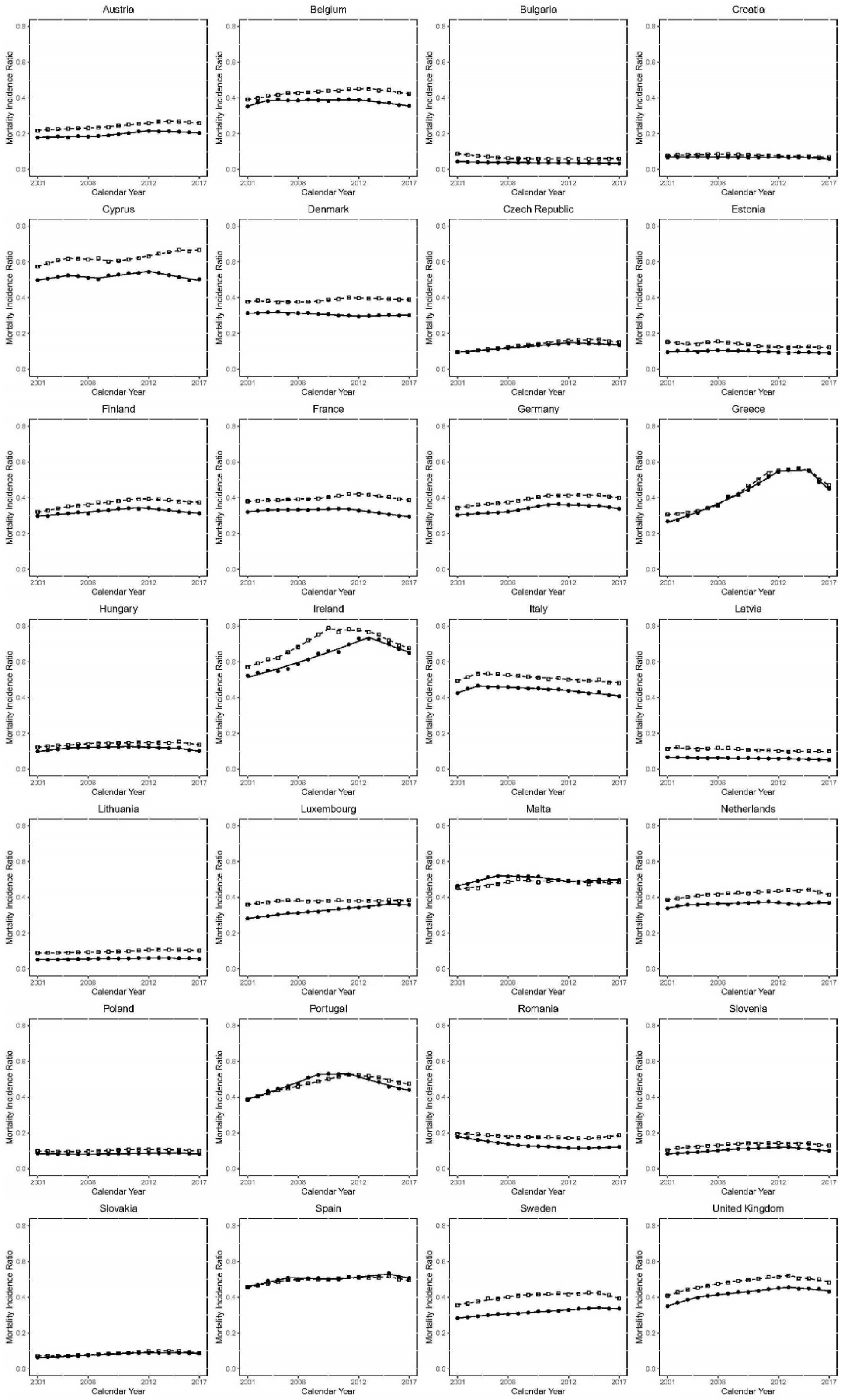

Supplementary figure S2:

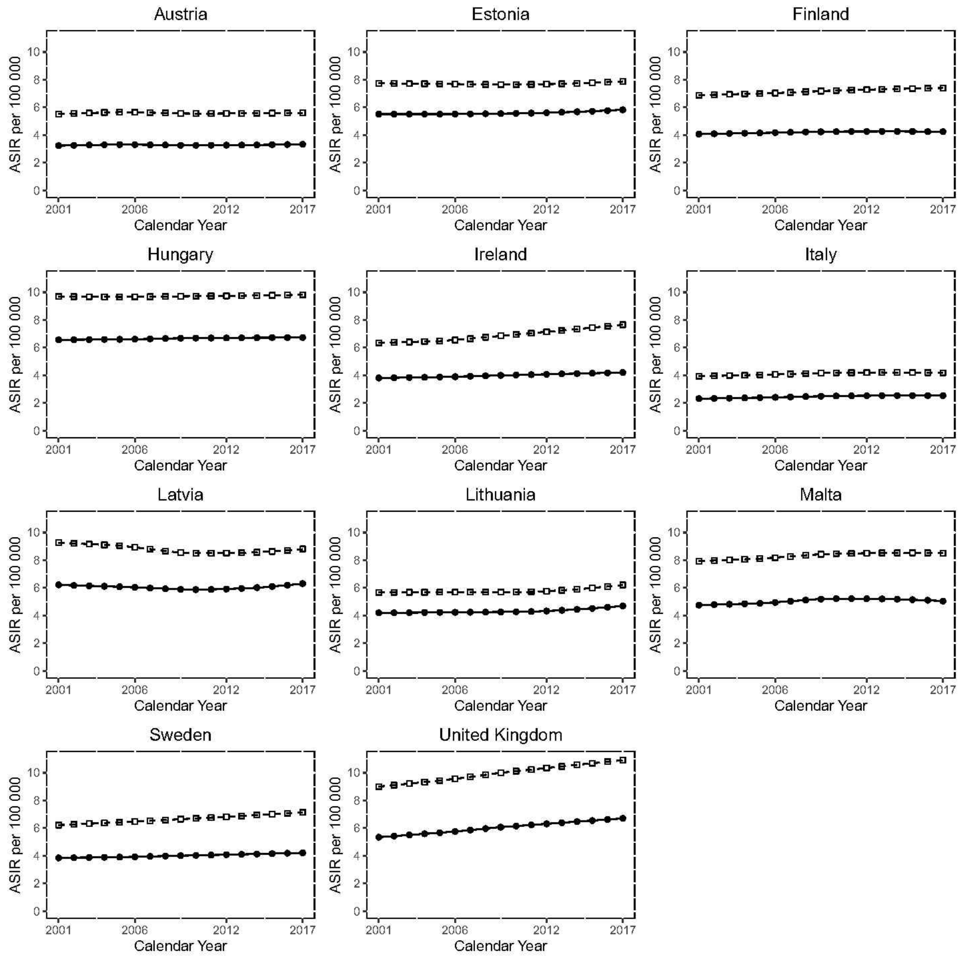

Supplementary figure S3:

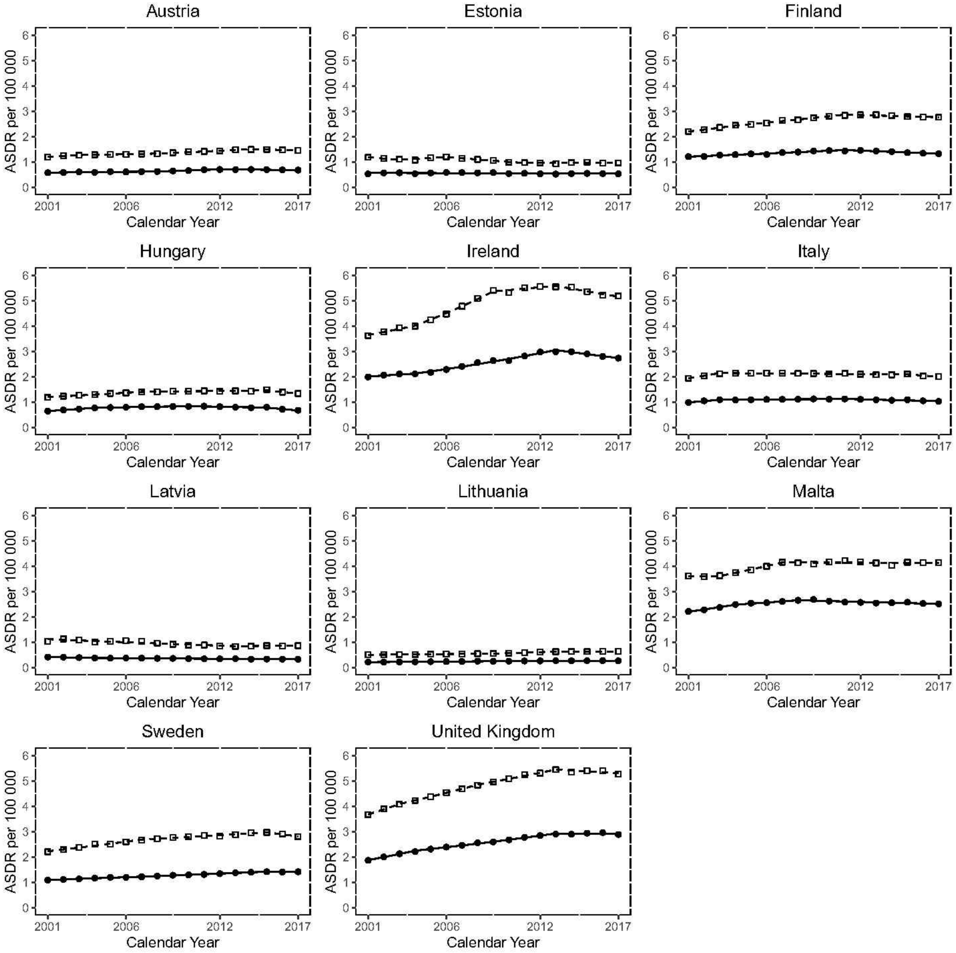

Supplementary figure S4:

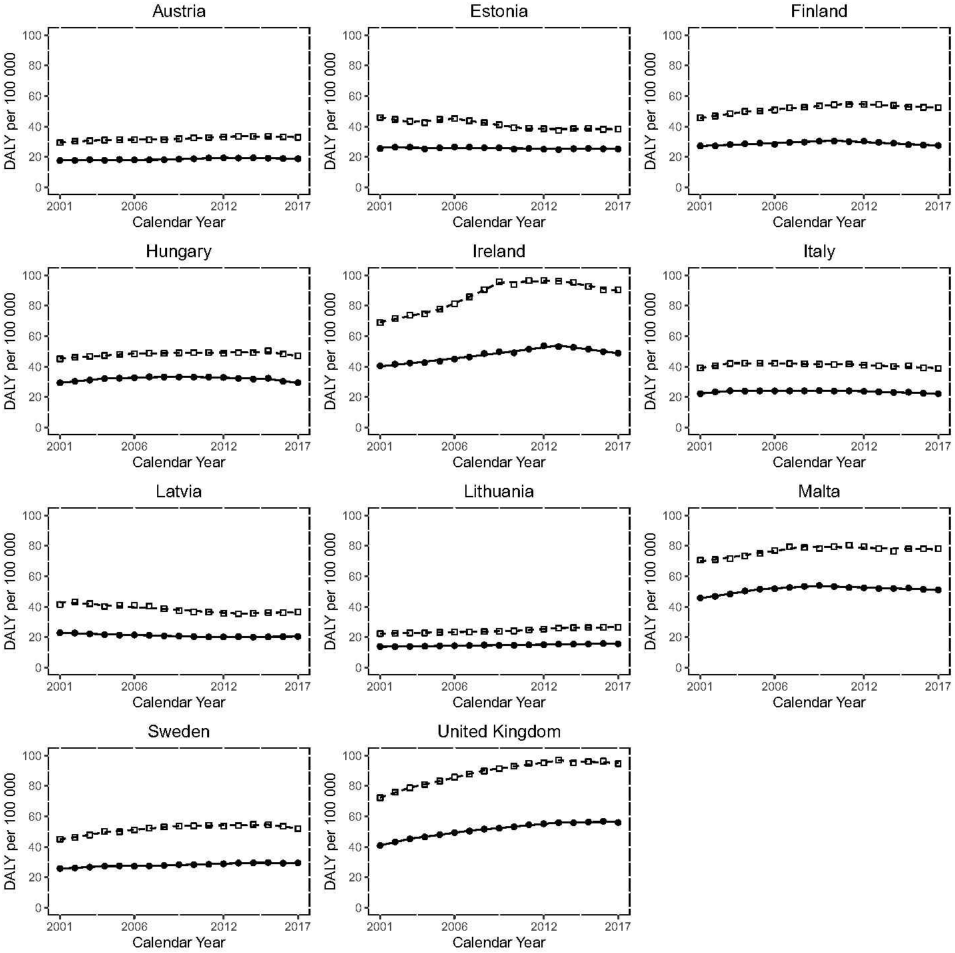

Supplementary figure S5:

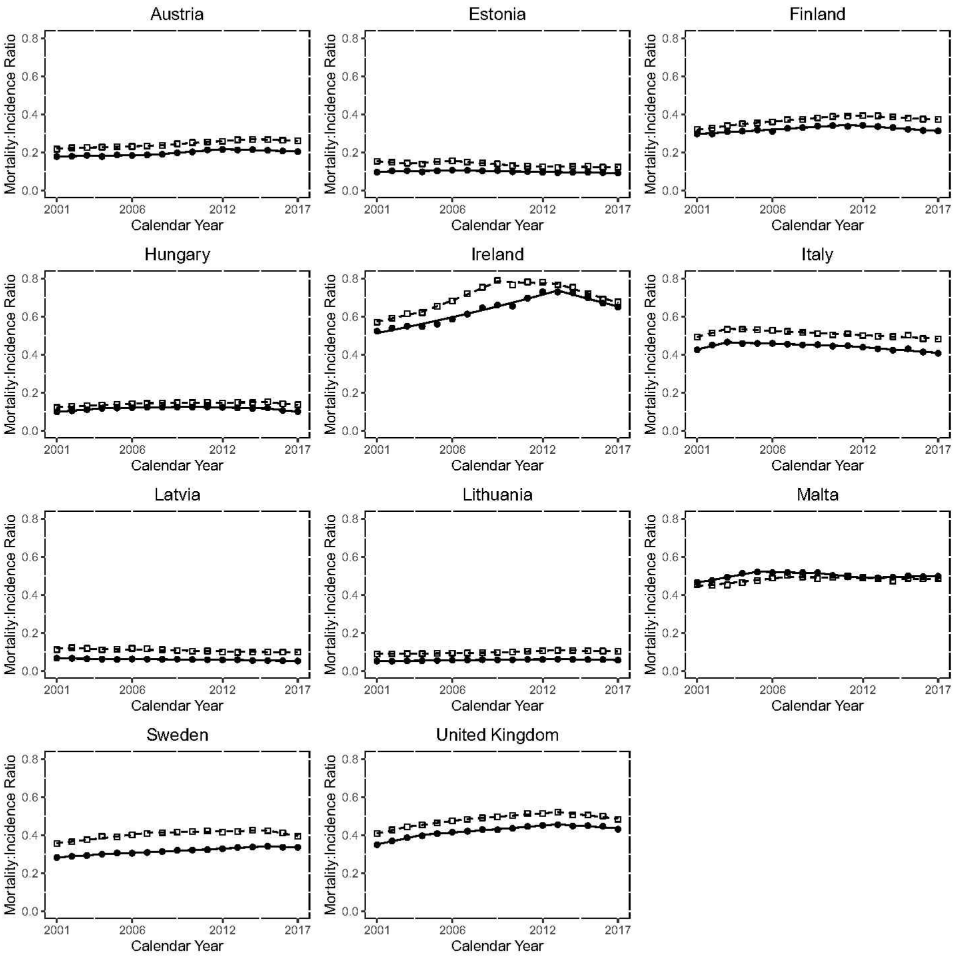

Supplementary table legends

Supplementary table S1: Joinpoint analysis for male incidence of interstitial lung diseases across Europe between 2001 and 2017. EAPC is estimated annual percentage change and CI is confidence interval of the EAPC

Supplementary table S2: Joinpoint analysis for female incidence of interstitial lung diseases across Europe between 2001 and 2017. EAPC is estimated annual percentage change and CI is confidence interval of the EAPC

Supplementary table S3: Joinpoint analysis for male mortality of interstitial lung diseases across Europe between 2001 and 2017. EAPC is estimated annual percentage change and CI is confidence interval of the EAPC

Supplementary table S4: Joinpoint analysis for male mortality of interstitial lung diseases across Europe between 2001 and 2017. EAPC is estimated annual percentage change and CI is confidence interval of the EAPC

Supplementary table S5: Joinpoint analysis of mortality-to-incidence ratio for men across European countries between 2001 and 2017. EAPC is estimated annual percentage change and CI is confidence interval of the EAPC.

Supplementary table S6: Joinpoint analysis of mortality-to-incidence ratio for women across European countries between 2001 and 2017. EAPC is estimated annual percentage change and CI is confidence interval of the EAPC.

Supplementary table S1:

	Trend 1		Trend 2		Trend 3		Trend 4	
	Years	EAPC (95% CI)	Years	EAPC (95% CI)	Years	EAPC (95% CI)	Years	EAPC (95% CI)
Austria	2001 – 2005	0.7 (0.6, 0.7)	2005 – 2010	-0.4 (-0.5, -0.3)	2010 – 2017	0.1 (0.1, 0.2)		
Belgium	2001 – 2005	0.5 (0.4, 0.5)	2005 – 2010	1.3 (1.3, 1.4)	2010 – 2017	0.9 (0.8, 0.9)		
Bulgaria	2001 – 2005	0.0 (-0.1, 0.0)	2005 – 2010	-0.4 (-0.5, -0.4)	2010 – 2014	0.0 (0.0, 0.1)	2014 – 2017	0.2 (0.2, 0.3)
Croatia	2001 – 2004	0.2 (0.2, 0.3)	2004 – 2011	0.2 (0.2, 0.2)	2011 – 2014	0.3 (0.3, 0.4)	2014 – 2017	0.4 (0.4, 0.5)
Cyprus	2001 – 2005	0.0 (-0.1, 0.0)	2005 – 2010	0.9 (0.8, 0.9)	2010 – 2014	-0.4 (-0.5, -0.3)	2014 – 2017	-1.3 (-1.4, -1.1)
Czech Republic	2001 – 2003	0.3 (0.2, 0.5)	2003 – 2006	0.5 (0.3, 0.7)	2006 – 2017	1.0 (1.0, 1.0)		
Denmark	2001 – 2005	0.3 (0.3, 0.3)	2005 – 2010	0.1 (0.1, 0.1)	2010 – 2013	0.2 (0.2, 0.3)	2013 - 2017	0.3 (0.2, 0.4)
Estonia	2001 – 2010	-0.1 (-0.2, -0.1)	2010 – 2013	0.2 (0.1. 0.4)	2013 – 2017	0.6 (0.5, 0.6)		`
Finland	2001 – 2006	0.4 (0.4-0.5)	2006 – 2010	0.7 (0.6, 0.7)	2010 – 2014	0.4 (0.3, 0.4)	2014 – 2017	0.3 (0.2-0.3)
France	2001 – 2006	0.3 (0.3, 0.3)	2006 – 2013	0.4 (0.4, 0.4)	2013 – 2017	0.5 (0.5, 0.5)		
Germany	2001 -2006	0.1 (0.1, 0.1)	2006 – 2012	0.7 (0.7, 0.7)	2012 – 2017	1.0 (1.0, 1.1)		
Greece	2001 – 2006	0.7 (0.6, 0.7)	2006 – 2010	2.0 (1.9, 2.1)	2010 – 2013	2.4 (2.2, 2.6)	2013 – 2017	3.0 (3.0, 3.1)
Hungary	2001 – 2005	-0.1 (-0.1, -0.1)	2005 – 2013	0.1 (0.1, 0.1)	2013 – 2017	0.2 (0.1, 0.2)		
Ireland	2001 – 2005	0.6 (0.4, 0.6)	2005 – 2017	1.4 (1.4, 1.4)				
Italy	2001 – 2006	0.6 (0.6, 0.7)	2006 – 2009	0.9 (0.7, 1.0)	2009 – 2013	0.3 (0.2, 0.3)	2013 – 2017	-0.1 (-0.2, -0.1)
Latvia	2001 – 2005	-0.6(-0.7, -0.4)	2005 – 2009	-1.5 (-1.7, -1.3)	2009 – 2013	-0.1 (-0.3, 0.1)	2013 – 2017	0.8 (0.7, 0.9)
Lithuania	2001 – 2011	0.1 (0.0, 0.1)	2011 – 2014	1.1 (0.9, 1.2)	2014 – 2017	1.7 (1.7, 1.8)		
Luxembourg	2001 – 2006	0.5 (0.5, 0.5)	2006 – 2009	0.6 (0.6, 0.6)	2009 – 2014	0.5 (0.4, 0.5)	2014 – 2017	0.4 (0.4, 0.4)
Malta	2001 – 2005	0.5 (0.5, 0.6)	2005 – 2009	1.0 (0.9, 1.1)	2009 – 2013	0.3 (0.2, 0.4)	2013 – 2017	-0.0 (-0.1, -0.0)
Netherlands	2001 – 2005	0.6 (0.5, 0.7)	2005 – 2011	1.7 (1.7, 1.8)	2011 – 2017	1.2 (1.2, 1.3)		
Poland	2001 – 2008	-0.1 (-0.1, -0.0)	2008 – 2011	0.0 (-0.0, 0.1)	2011 – 2014	0.3 (0.3, 0.4)	2014 – 2017	0.6 (0.6, 0.6)
Portugal	2001 – 2006	0.5 (0.5, 0.5)	2006 – 2009	1.0 (0.9, 1.0)	2009 – 2017	0.6 (0.6, 0.6)		
Romania	2001 – 2004	-1.1 (-1.2, -1.1)	2004 – 2011	-0.5 (-0.5, -0.5)	2011 – 2014	-1.0 (-1.1, -0.8)	2014 – 2017	-1.3 (-1.4, -1.3)
Slovenia	2001 – 2006	0.3 (0.3, 0.3)	2006 – 2009	0.8 (0.7, 0.8)	2009 – 2017	0.4 (0.4, 0.4)		
Slovakia	2001 – 2006	0.3 (0.2, 0.3)	2006 – 2009	0.5 (0.5, 0.6)	2013 – 2017	0.4 (0.4, 0.5)		
Spain	2001 – 2006	0.5 (0.5, 0.5)	2006 – 2009	1.0 (0.9, 1.0)	2009 – 2013	0.5 (0.5, 0.5)		
Sweden	2001 – 2006	0.8 (0.8, 0.8)	2006 – 2014	0.9 (0.9, 0.9)	2014 – 2017	0.9 (0.9, 1.0)		
United Kingdom	2001 – 2006	1.2 (1.2, 1.2)	2006 – 2009	1.5 (1.5, 1.6)	2009 – 2014	1.1 (1.1, 1.1)	2014 – 2017	1.0 (1.0, 1.1)

Supplementary table S2:

	Trend 1		Trend 2		Trend 3		Trend 4	
	Years	EAPC (95% CI)	Years	EAPC (95% CI)	Years	EAPC (95% CI)	Years	EAPC (95% CI)
Austria	2001 – 2005	0.6 (0.5, 0.6)	2005 – 2010	-0.4 (-0.4, -0.3)	2010 – 2014	0.2 (0.2, 0.3)	2014 – 2017	0.5 (0.4, 0.5
Belgium	2001 – 2005	0.5 (0.5, 0.6)	2005 – 2009	1.3 (1.2, 1.4)	2009 – 2012	0.7 (0.4, 09)	2012 – 2017	0.3 (0.3, 0.4)
Bulgaria	2001 – 2004	0.4 (0.4, 0.5)	2004 – 2011	0.2 (0.2, 0.2)	2011 – 2014	0.7 (0.6, 0.8)	2014 – 2017	1.1 (1.0, 1.1)
Croatia	2001 – 2006	0.1 (0.0, 0.1)	2006 – 2011	0.2 (0.2, 0.2)	2011 – 2014	0.7 (0.7, 0.8)	2014 – 2017	1.1 (1.1, 1.1)
Cyprus	2001 – 2011	-0.2 (-0.2, -0.1)	2011 – 2014	-1.0 (-1.4, -0.6)	2014 – 2017	-1.6 (-1.8, -1.4)		
Czech Republic	2001 – 2006	0.4 (0.4, 0.4)	2006 – 2009	0.8 (0.7, 0.8)	2009 – 2017	0.6 (0.6, 0.6)		
Denmark	2001 -2005	0.6 (0.6, 0.7)	2005 – 2017	0.0 (0.0, 0.0)				
Estonia	2001 – 2006	0.0 (-0.0, 0.0)	2006 – 2011	0.2 (0.2, 0.3)	2011 – 2014	0.6 (0.5, 0.7)	2014 – 2017	0.8 (0.8, 0.9)
Finland	2001 – 2010	0.5 (0.5, 0.5)	2010 – 2014	0.1 (-0.0, 0.1)	2014 – 2017	-0.2 (-0.3, -0.1)		
France	2001 – 2004	0.5 (0.5, 0.5)	2004 – 2011	0.3 (0.3, 0.3)	2011 – 2014	0.4 (0.3, 0.4)	2014 – 2017	0.4 (0.4, 0.4)
Germany	2001 – 2008	0.4 (0.4, 0.4)	2008 – 2011	0.6 (0.5, 0.7)	2011 – 2014	0.8 (0.7, 0.9)	2014 – 2017	1.0 (1.0,1.1)
Greece	2001 -2003	0.5 (0.0, 0.9)	2003 – 2006	1.0 (0.6, 1.4)	2006 – 2014	2.6 (2.5, 2.6)	2014 – 2017	2.8 (2.6, 3.0)
Hungary	2001 – 2006	0.1 (0.1, 0.1)	2006 – 2009	0.4 (0.3, 0.4)	2009 – 2014	0.1 (0.1, 0.1)	2014 – 2017	0.1 (0.1, 0.1)
Ireland	2001 – 2005	0.4 (0.4, 0.4)	2005 – 2009	0.8 (0.8, 0.8)	2009 – 2017	0.6 (0.6, 0.7)		
Italy	2001 – 2005	0.7 (0.7, 0.8)	2005 – 2010	1.0 (1.0, 1.1)	2010 – 2014	0.3 (0.2, 0.3)	2014 – 2017	-0.1 (-0.1, -0.0)
Latvia	2001 – 2005	-0.5 (-0.6, -0.5)	2005 – 2010	-0.8 (-0.9, -0.7)	2010 – 2014	0.6 (0.5, 0.8)	2014 – 2017	1.6 (1.5, 1.8)
Lithuania	2001 – 2011	0.2 (0.2, 0.2)	2011 – 2014	1.2 (1.1, 1.4)	2014 – 2017	1.8 (1.8, 1.9)		
Luxembourg	2001 – 2006	0.8 (0.7, 0.8)	2006 – 2009	1.2 (1.2, 1.2)	2009 – 2017	1.1 (1.1, 1.1)		
Malta	2001 – 2005	0.6 (0.5, 0.8)	2005 – 2009	1.6 (1.4,1.8)	2009 – 2013	0.1 (-0.1, 0.3)	2013 – 2017	-0.8 (-0.9, -0.7)
Netherlands	2001 – 2005	0.6 (0.6, 0.6)	2005 – 2010	1.2 (1.2, 1.2)	2010 – 2014	0.8 (0.8, 0.9)	2014 – 2017	0.7 (0.7, 0.7)
Poland	2001 - 2005	-0.1 (-0.1, -0.2)	2005 – 2012	0.2 (0.1, 0.2)	2012 – 2017	0.3 (0.3, 0.3)		
Portugal	2001 – 2005	0.4 (0.4, 0.4)	2005 – 2010	1.0 (1.0, 1.1)	2010 – 2014	0.5 (0.5, 0.5)	2014 – 2017	0.3 (0.3, 0.4)
Romania	2001 – 2003	-1.6 (-1.6, -1.6)	2003 – 2008	-1.4 (-1.4, -1.4)	2008 – 2012	-1.4 (-1.4, -1.4)	2012 – 2017	-1.5 (-1.5, -1.5)
Slovenia	2001 – 2003	0.2 (-0.0, 0.4)	2003 – 2006	0.4 (0.2, 0.6)	2006 – 2010	1.1 (1.0, 1.2)	2010 – 2017	0.7 (0.6, 0.7)
Slovakia	2001 – 2006	0.3 (0.3, 03)	2006 – 2010	0.4 (0.3, 0.4)	2010 – 2014	0.1 (0.1, 0.2)	2014 – 2017	-0.0 (-0.1, 0.0)
Spain	2001 – 2005	0.5 (0.5, 0.6)	2005 – 2010	1.5 (1.4, 1.5)	2010 – 2014	0.0 (-0.1, 0.1)	2014 – 2017	-0.8 (-0.9, -0.7)
Sweden	2001 – 2006	0.4 (0.4, 0.4)	2006 – 2009	0.7 (0.7, 0.8)	2009 – 2013	0.6 (0.6, 0.6)	2013 – 2017	0.7 (0.6, 0.7)
United Kingdom	2001 – 2006	1.5 (1.5, 1.5)	2006 – 2009	1.8 (1.7, 1.9)	2009 – 2014	1.3 (1.2, 1.3)	2014 – 2017	1.2 (1.1, 1.2)

Supplementary table S3:

	Trend 1		Trend 2		Trend 3		Trend 4	
	Years	EAPC (95% CI)	Years	EAPC (95% CI)	Years	EAPC (95% CI)	Years	EAPC (95% CI)
Austria	2001 – 2003	2.9 (1.4, 4.4)	2003 – 2008	0.9 (0.4, 1.4)	2008 – 2014	2.1 (1.7, 2.4)	2014 – 2017	-0.9 (-1.7, -0.2)
Belgium	2001 – 2012	2.3 (2.1, 2.4)	2012 – 2017	-0.5 (-1.1, -0.0)				
Bulgaria	2001 – 2006	-6.7 (-7.3, -6.2)	2006 – 2009	-2.4 (-5.0, 0.3)	2009 – 2017	0.3 (-0.0, 0.6)		
Croatia	2001 – 2006	1.9 (0.5, 3.4)	2006 – 2017	-1.8 (-2.2, -1.4)				
Cyprus	2001 – 2003	3.3 (0.5, 6.1)	2003 – 2009	0.4 (-0.2, 1.0)	2009 – 2014	1.5 (0.6, 2.3)	2014 – 2017	-0.8 (-2.2, 0.5)
Czech Republic	2001 – 2012	5.7 (5.4, 6.0)	2012 – 2015	1.8 (-2.1, 5.7)	2015 – 2017	-3.9 (-7.5, -0.1)		
Denmark	2001 – 2007	-0.0 (-0.5, 0.4)	2007 – 2011	1.7 (0.3, 3.0)	2011 – 2017	-0.2 (-0.6, 0.3)		
Estonia	2001 – 2003	-4.6 (-11.2, 2.6)	2003 – 2006	3.4 (-3.8, 11.1)	2006 – 2011	-4.3 (-6.4, -2.1)	2011 – 2017	0.1 (-1.1, 1.3)
Finland	2001 – 2004	3.4 (2.5, 4.4)	2004 – 2011	2.4 (2.1, 2.7)	2011 – 2017	-0.6 (-0.9, -0.3)		
France	2001 – 2007	0.8 (0.6, 1.0)	2007 – 2012	2.0 (1.6, 2.3)	2012 – 2017	-1.3 (-1.5, -1.1)		
Germany	2001 – 2006	1.8 (1.2, 2.3)	2006 – 2010	3.1 (1.9, 4.3)	2010 – 2015	-0.8 (-3.1, 1.6)	2015 – 2017	-0.8 (-3.1, 1.6)
Greece	2001 – 2005	3.0 (1.0, 5.0)	2005 – 2011	10.4 (8.9, 12.0)	2011 – 2015	3.5 (0.4, 6.7)	2015 – 2017	-5.8 (-11.4, 0.2)
Hungary	2001 – 2007	2.7 (2.3, 3.1)	2007 – 2015	0.5 (0.2, 0.8)	2015 – 2017	-4.5 (-6.6, -2.4)		
Ireland	2001 – 2004	3.3 (2.0, 4.5)	2004 – 2009	6.0 (5.1, 6.8)	2009 – 2013	1.0 (-0.2, 2.3)	2013 – 2017	-2.0 (-2.7, -1.2)
Italy	2001 – 2003	5.2 (3.3, 7.2)	2003 – 2015	-0.2 (-0.3, -0.1)	2015 – 2017	-2.1 (-3.9, -0.3)		
Latvia	2001 – 2013	-2.3 (-3.0, -1.7)	2013 – 2017	0.6 (-3.0, 4.3)				
Lithuania	2001 – 2009	1.1 (0.9, 1.3)	2009 – 2013	3.4 (2.6, 4.2)	2013 – 2017	0.3 (-0.2, 0.8)		
Luxembourg	2001 – 2004	2.4 (1.3, 3.5)	2004 – 2017	0.5 (0.4, 0.6)				
Malta	2001 – 2003	0.1 (-3.8, 4.2)	2003 – 2007	3.5 (1.5, 5.6)	2007 – 2017	-0.0 (-0.3, 0.3)		
Netherlands	2001 – 2012	2.4 (2.3, 2.5)	2012 – 2015	1.6 (-0.1, 3.4)	2015 – 2017	-1.9 (-3.6, -0.1)		
Poland	2001 – 2005	-1.0 (-1.3, -0.6)	2005 – 2010	2.5 (2.2, 2.9)	2010 – 2015	-0.0 (-0.4, 0.3)	2015 – 2017	-2.7 (-3.7, -1.6)
Portugal	2001 – 2003	5.1 (3.2, 7.1)	2003 – 2011	3.6 (3.3, 3.9)	2011 – 2014	-0.7 (-2.5, 1.1)	2014 – 2017	-1.9 (-2.8, -1.0)
Romania	2001 – 2007	-2.5 (-2.8, -2.2)	2007 – 2010	-0.7 (-2.4, 1.1)	2010 – 2014	-1.8 (-2.7, -0.9)	2014 -2017	1.8 (0.9, 2.7)
Slovenia	2001 – 2003	7.2 (3.2, 11.5)	2003 – 2009	3.4 (2.5, 4.3)	2009 – 2015	0.2 (-0.7, 1.0)	2015 – 2017	-4.1 (-7.7, -0.3)
Slovakia	2001 – 2004	0.6 (-0.9, 2.2)	2004 – 2012	4.0 (3.5, 4.4)	2012 – 2015	1.0 (-2.1, 4.2)	2015 – 2017	-5.1 (-8.1, -2.1
Spain	2001 – 2005	2.6 (2.2, 3.0)	2005 – 2011	1.2 (0.9, 1.5)	2011 – 2015	0.6 (-0.1, 1.2)	2015 – 2017	-1.5 (-2.7, -0.3)
Sweden	2001 – 2007	3.2 (2.6, 3.8)	2007 – 2015	1.3 (0.8, 1.7)	2015 – 2017	-2.7 (-6.0, 0.7)		
United Kingdom	2001 – 2003	5.4 (2.4, 8.5)	2003 – 2008	3.5 (2.5, 4.4)	2008 – 2013	2.4 (1.4, 3.3)	2013 – 2017	-0.6 (-1.6, 0.3)

Supplementary table S4:

	Trend 1		Trend 2		Trend 3		Trend 4	
	Years	EAPC (95% CI)	Years	EAPC (95% CI)	Years	EAPC (95% CI)	Years	EAPC (95% CI)
Austria	2001 – 2008	1.0 (0.4, 1.6)	2008 – 2012	3.2 (1.1, 5.4)	2012 – 2017	-0.7 (-1.7, 0.2)		
Belgium	2001 – 2003	4.9 (1.9, 7.9)	2003 – 2012	1.1 (0.8, 1.4)	2012 – 2017	-1.6 (-2.3, -1.0)		
Bulgaria	2001 – 2003	-4.6 (-6.9, -2.2)	2003 – 2017	-0.7 (-0.8, -0.6)				
Croatia	2001 – 2015	0.2 (-0.2, 0.5)	2015 – 2017	-5.9 (-12.7, 1.5)				
Cyprus	2001 – 2012	0.5 (0.2, 0.8)	2012 – 2017	-3.0 (-4.1, -2.0)				
Czech Republic	2001 – 2012	4.7 (4.4, 5.0)	2012 – 2017	-1.1 (-2.1, -0.0)				
Denmark	2001 – 2004	1.4 (-0.2, 3.1)	2004 – 2012	-0.8 (-1.2, -0.4)	2012 – 2017	0.4 (-0.3, 1.1)		
Estonia	2001 – 2017	-0.3 (-0.7, 0.0)						
Finland	2001 – 2011	2.0 (1.7, 2.3)	2011 – 2017	-1.7 (-2.3, -1.1)				
France	2001 – 2003	2.2 (0.7, 3.7)	2003 – 2011	0.5 (0.3, 0.7)	2011 – 2017	-1.9 (-2.2, -1.7)		
Germany	2001 – 2006	1.6 (1.1, 2.2)	2006 – 2011	3.2 (2.4, 4.0)	2011 – 2017	-0.2 (-0.8, 0.1)		
Greece	2001 – 2004	6.4 (2.9, 10.0)	2004 – 2012	9.6 (8.6, 10.6)	2012 – 2015	2.9 (-3.7, 10.1)	2015 – 2017	-7.8 (-13.8, -1.3)
Hungary	2001 – 2004	6.3 (5.0, 7.6)	2004 – 2010	1.3 (0.8, 1.9)	2010 – 2015	-1.4 (-2.2, -0.6)	2015 – 2017	-7.3 (-9.5, -5.1)
Ireland	2001 – 2004	1.9 (-0.6, 4.4)	2004 – 2013	4.1 (3.6, 4.7)	2013 – 2017	-2.7 (-4.2 -1.2)		
Italy	2001 – 2003	5.0 (1.7, 8.4)	2003 – 2011	0.4 (-0.0, 0.8)	2011 – 2017	-1.3 (-1.9, -0.8)		
Latvia	2001 – 2004	-3.1 (-5.1, -0.9)	2004 – 2017	-1.1 (-1.4, -0.9)				
Lithuania	2001 – 2013	1.9 (1.6, 2.2)	2013 – 2017	-0.1 (-1.7, 1.6)				
Luxembourg	2001 – 2015	2.8 (2.7, 2.9)	2015 – 2017	0.5 (-1.9, 2.9)				
Malta	2001 – 2004	4.1 (2.5, 5.6)	2004 – 2008	1.7 (0.2, 3.2)	2008 – 2017	-0.6 (-0.9, -0.3)		
Netherlands	2001 – 2010	1.7 (1.3, 2.1)	2010 – 2017	0.6 (0.1, 1.2)				
Poland	2001 – 2005	-1.4 (-2.2, -0.6)	2005 – 2015	1.2 (1.0, 1.5)	2015 – 2017	-4.1 (-6.5, -1.7)		
Portugal	2001 – 2008	5.2 (4.5, 5.8)	2008 – 2011	1.2 (-3.4, 6.1)	2011 – 2017	-2.8 (-3.6, -2.0)		
Romania	2001 – 2007	-6.5 (-6.9, -6.1)	2007 – 2013	-3.6 (-4.1, -3.0)	2013 – 2017	-0.0 (-0.9, 0.8)		
Slovenia	2001 – 2008	4.9 (4.3, 5.4)	2008 – 2013	2.6 (1.4, 3.8)	2013 – 2017	-4.3 (-5.4, -3.1)		
Slovakia	2001 – 2010	4.4 (4.1, 4.6)	2010 – 2015	0.6 (-0.2, 1.4)	2015 – 2017	-3.1 (-5.5, -0.6)		
Spain	2001 – 2005	3.2 (2.3, 4.1)	2005 – 2015	1.1 (0.8, 1.4)	2015 – 2017	-3.4 (-6.1, -0.6)		
Sweden	2001 – 2015	1.9 (1.8, 2.0)	2015 – 2017	-0.3 (-2.3, 1.6)				
United Kingdom	2001 – 2004	6.2 (4.8, 7.6)	2004 – 2013	3.0 (2.7, 3.3)	2013 – 2017	-0.1 (-0.9, 0.7)		

Supplementary table S5:

	Trend 1		Trend 2		Trend 3		Trend 4	
	Years	EAPC (95% CI)	Years	EAPC (95% CI)	Years	EAPC (95% CI)	Years	EAPC (95% CI)
Austria	2001 – 2007	1.1 (0.6, 1.5)	2007 – 2014	2.1 (1.6, 2.5)	2014 – 2017	-1.2 (-2.5, 0.1)		
Belgium	2001 – 2005	2.1 (1.4, 2.8)	2005 – 2013	0.8 (0.5, 1.1)	2013 – 2017	-1.7 (-2.3, -1.0)		
Bulgaria	2001 – 2006	-6.7 (-7.2, -6.1)	2006 – 2009	-1.7 (-4.3, 0.9)	2009 – 2017	0.2 (-0.1, 0.5)		
Croatia	2001 – 2006	1.8 (0.4, 3.2)	2006 – 2017	-2.1 (-2.5, -1.7)				
Cyprus	2001 – 2004	2.6 (1.2, 4.1)	2004 – 2009	-0.6 (-1.5, 0.3)	2009 – 2015	1.6 (1.0, 2.2)	2015 – 2017	-0.0 (-2.8, 2.9)
Czech Republic	2001 – 2011	5.1 (4.7, 5.4)	2011 – 2015	1.6 (-0.8, 4.0)	2015 – 2017	-5.2 (-9.5, -0.7)		
Denmark	2001 – 2007	-0.2 (-0.7, 0.2)	2007 – 2011	1.6 (0.3, 2.9)	2011 – 2017	-0.5 (-0.9, -0.0)		
Estonia	2001 – 2003	-4.4 (-11.2, 2.9)	2003 – 2006	3.5 (-3.8, 11.5)	2006 – 2011	-4.2 (-6.4, -1.9)	2011 – 2017	-0.4 (-1.6, 0.8)
Finland	2001 – 2004	3.1 (2.1, 4.0)	2004 – 2011	1.8 (1.5, 2.1)	2011 – 2017	-0.9 (-1.2, -0.6)		
France	2001 – 2008	0.6 (0.5, 0.7)	2008 – 2012	1.7 (1.2, 2.2)	2012 – 2017	-1.8 (-2.1, -1.6)		
Germany	2001 – 2007	1.7 (1.3, 2.1)	2007 – 2010	2.6 (0.1, 5.2)	2010 – 2015	0.1 (-0.7, 0.9)	2015 – 2017	-1.9 (-4.3, 0.6)
Greece	2001 – 2005	2.5 (0.5, 4.5)	2005 – 2011	8.4 (6.9, 10.0)	2011 – 2015	0.7 (-2.5, 3.9)	2015 – 2017	-8.7 (-14.2, -2.8)
Hungary	2001 – 2007	2.7 (2.3, 3.1)	2007 – 2015	0.4 (0.1, 0.7)	2015 – 2017	-4.7 (-6.8, -2.5)		
Ireland	2001 – 2004	2.9 (1.7, 4.2)	2004 – 2009	4.6 (3.8, 5.4)	2009 – 2013	-0.4 (-1.6, 0.8)	2013 – 2017	-3.3 (-4.1, -2.6)
Italy	2001 – 2003	4.3 (1.6, 7.1)	2003 – 2017	-0.7 (-0.9, -0.6)				
Latvia	2001 – 2017	-1.4 (-1.8, -1.0)						
Lithuania	2001 – 2004	0.6 (-0.0, 1.3)	2004 – 2009	1.3 (0.9, 1.7)	2009 – 2013	2.8 (2.2, 35)	2013 – 2017	-1.3 (-1.7, -1.0)
Luxembourg	2001 – 2004	1.8 (0.7, 3.0)	2004 – 2017	0.0 (-0.1, 0.2)				
Malta	2001 – 2007	1.8 (1.0, 2.5)	2007 – 2017	-0.2 (-0.6, 0.1)				
Netherlands	2001 – 2005	1.8 (1.1, 2.5)	2005 – 2015	0.7 (0.5, 0.9)	2015 – 2017	-3.2 (-5.3, -1.1)		
Poland	2001 – 2005	-0.9 (-1.3, -0.5)	2005 – 2010	2.6 (2.2, 3.0)	2010 – 2015	-0.4 (-0.7, 0.0)	2015 – 2017	-3.3 (-4.5, -2.2)
Portugal	2001 – 2004	4.2 (3.4, 4.9)	2004 – 2011	2.7 (2.5, 3.0)	2011 – 2014	-1.3 (-2.7, 0.2)	2014 – 2017	-2.5 (-3.2, -1.8)
Romania	2001 – 2007	-1.6 (-1.9, -1.3)	2007 – 2014	-0.7 (-1.0, -0.4)	2014 – 2017	3.0 (2.0, 4.1)		
Slovenia	2001 – 2003	7.2 (3.1, 11.4)	2003 – 2009	2.8 (1.9, 3.7)	2009 – 2015	-0.3 (-1.2, 0.6)	2015 – 2017	-4.4 (-8.0, -0.6)
Slovakia	2001 – 2004	0.7 (-0.9, 2.3)	2004 – 2014	3.3 (3.0, 3.6)	2014 – 2017	-4.6 (-6.1, -3.0)		
Spain	2001 – 2005	2.2 (1.6, 2.7)	2005 – 2015	0.3 (0.2, 0.5)	2015 – 2017	-2.0 (-3.6, -0.3)		
Sweden	2001 – 2007	2.4 (1.8, 3.0)	2007 – 2015	0.4 (-0.1, 0.8)	2015 – 2017	-3.6 (-6.9, -0.3)		
United Kingdom	2001 – 2003	4.2 (1.2, 7.2)	2003 – 2007	2.2 (0.8, 3.7)	2007 – 2013	1.2 (0.6, 1.9)	2013 – 2017	-1.7 (-2.6, -0.8)

Supplementary table S6:

	Trend 1		Trend 2		Trend 3		Trend 4	
	Years	EAPC (95% CI)	Years	EAPC (95% CI)	Years	EAPC (95% CI)	Years	EAPC (95% CI)
Austria	2001 – 2007	0.6 (-0.1, 1.3)	2007 – 2012	3.1 (1.8, 4.5)	2012 – 2017	-1.1 (-2.0, -0.2)		
Belgium	2001 – 2003	4.5 (1.5, 7.6)	2003 – 2012	0.1 (-0.2, 0.4)	2012 – 2017	-1.9 (-2.5, -1.3)		
Bulgaria	2001 – 2004	-3.9 (-5.0, -2.7)	2004 – 2011	-0.6 (-1.0, -0.2)	2011 – 2017	-1.7 (-2.1, -1.3)		
Croatia	2001 – 2015	-0.1 (-0.5, 0.2)	2015 – 2017	-7.6 (-14.6, -0.1)				
Cyprus	2001 – 2004	1.7 (0.0, 3.4)	2004 – 2007	-0.9 (-4.1, 2.4)	2007 – 2012	1.4 (0.4, 2.5)		
Czech Republic	2001 – 2012	4.1 (3.8, 4.4)	2012 – 2017	-1.6 (-2.7, -0.6)				
Denmark	2001 – 2004	0.6 (-1.0, 2.3)	2004 – 2012	-0.9 (-1.3, -0.5)	2012 – 2017	0.4 (-0.4, 1.1)		
Estonia	2001 – 2006	1.4 (-0.5, 3.3)	2006 – 2017	-1.3 (-1.9, -0.7)				
Finland	2001 – 2011	1.5 (1.2, 1.8)	2011 – 2017	-1.6 (-2.2, -1.0)				
France	2001 – 2003	1.5 (0.1, 3.0)	2003 - 2011	0.2 (0.0, 0.4)	2011 – 2017	-2.3 (-2.5, -2.1)		
Germany	2001 – 2006	1.1 (0.7, 1.6)	2006 – 2010	3.1 (2.0, 4.2)	2010 – 2015	-0.4 (-1.1, 0.2)	2015 – 2017	-2.4 (-4.5, -0.3)
Greece	2001 – 2012	7.0 (6.6, 7.4)	2012 – 2015	0.3 (-5.7, 6.6)	2015 – 2017	-10.3 (-15.6, -4.6)		
Hungary	2001 – 2004	6.2 (4.9, 7.6)	2004 – 2010	1.1 (0.5, 1.6)	2010 – 2015	-1.5 (-2.3, -0.7)	2015 – 2017	-7.4 (-9.7, -5.0)
Ireland	2001 – 2013	3.1 (2.7, 3.4)	2013 – 2017	-3.0 (-4.8, -1.1)				
Italy	2001 – 2003	4.4 (1.2, 7.6)	2003 – 2011	-0.5 (-1.0, -0.1)	2011 – 2017	-1.4 (-1.9, -0.9)		
Latvia	2001 – 2013	-1.0 (-1.4, -0.7)	2013 – 2017	-2.6 (-4.3, -0.9)				
Lithuania	2001 – 2013	1.6 (1.2, 1.9)	2013 – 2017	-2.1 (-3.7, -0.3)				
Luxembourg	2001 – 2004	2.5 (1.2, 3.7)	2004 – 2015	1.6 (1.4, 1.8)	2015 – 2017	-0.5 (-2.9, 1.9)		
Malta	2001 – 2005	2.9 (2.2, 3.7)	2005 – 2009	-0.4 (-1.6, 0.8)	2009 – 2012	-1.6 (-3.9, 0.8)	2012 – 2017	0.4 (-0.2, 0.9)
Netherlands	2001 – 2003	2.7 (0.0, 5.4)	2003 – 2011	0.5 (0.2, 0.9)	2011 – 2014	-1.0 (-3.6, 1.6)	2014 – 2017	0.9 (-0.5, 2.2)
Poland	2001 – 2005	-1.3 (-2.1, -0.5)	2005 – 2015	1.1 (0.8, 1.3)	2015 – 2017	-4.6 (-7.0, -2.0)		
Portugal	2001 – 2008	4.4 (3.8, 5.1)	2008 – 2011	0.2 (-4.5, 5.1)	2011 – 2017	-3.2 (-4.0, -2.4)		
Romania	2001 – 2007	-5.1 (-5.5, -4.7)	2007 – 2013	-2.2 (-2.8, -1.6)	2013 – 2017	1.4 (0.6, 2.3)		
Slovenia	2001 – 2008	4.3 (3.8, 4.7(	2008 – 2013	1.7 (0.5, 2.8)	2013 – 2017	-4.8 (-5.8, -3.7)		
Slovakia	2001 – 2010	4.0 (3.8, 4.3)	2010 – 2015	0.5 (-0.3, 1.3)	2015 – 2017	-3.0 (-5.3, -0.5)		
Spain	2001 – 2005	2.6 (1.7, 3.5)	2005 – 2010	-0.3 (-1.2, 0.6)	2010 – 2015	1.1 (0.2, 2.0)	2015 – 2017	-2.3 (-5.0, 0.5)
Sweden	2001 – 2004	2.0 (1.0, 2.9)	2004 – 2015	1.2 (1.0, 1.3)	2015 – 2017	-0.9 (-2.7, 1.0)		
United Kingdom	2001 – 2004	4.6 (3.2, 6.0)	2004 – 2013	1.4 (1.1, 1.7)	2013 – 2017	-1.2 (-2.0, -0.3)		
